# Supplementary material for: Quantifying the impact of an invasive hornet on Bombus terrestris colonies
Source: Commun Biol. 2023 Oct 5;6:990. doi: 10.1038/s42003-023-05329-5 (PMC10556089; doi:10.1038/s42003-023-05329-5)
Supplement: Supplementary file 2 — Supplementary Information [file 42003_2023_5329_MOESM2_ESM.pdf]

Quantifying the Impact of an Invasive Hornet on *Bombus terrestris* Colonies

Thomas A. O'Shea-Wheller, Robin J. Curtis, Peter J. Kennedy, Ellen K. J. Groom, Juliette Poidatz, David S. Raffle, Sandra V. Rojas-Nossa, Carolina Bartolomé, Damián Dasilva-Martins, Xulio Maside, Salustiano Mato, Juliet L. Osborne

Supplementary Information

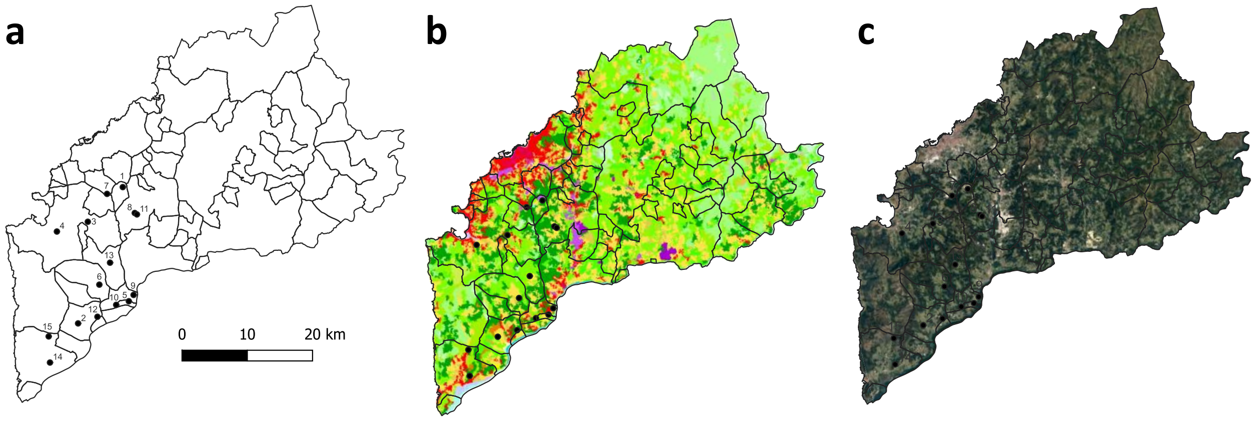

Fig. S1. The location of field sites within the counties of O Condado, O Baixo Miño, A Paradanta, and Vigo, in the province of Pontevedra, Spain. Choropleth maps show (a) site IDs and locations; (b) land cover classes; and (c) corresponding satellite imagery. Solid lines designate the boundaries of administrative municipalities, points indicate field site locations, and numbers denote site IDs. Maps were generated in QGIS (release v. 3.26.3), using the CORINE 2018 land cover dataset at a resolution of $100m^2$, and the Esri 2022 satellite imagery WGS84 basemap at a resolution of $\leq$1m.

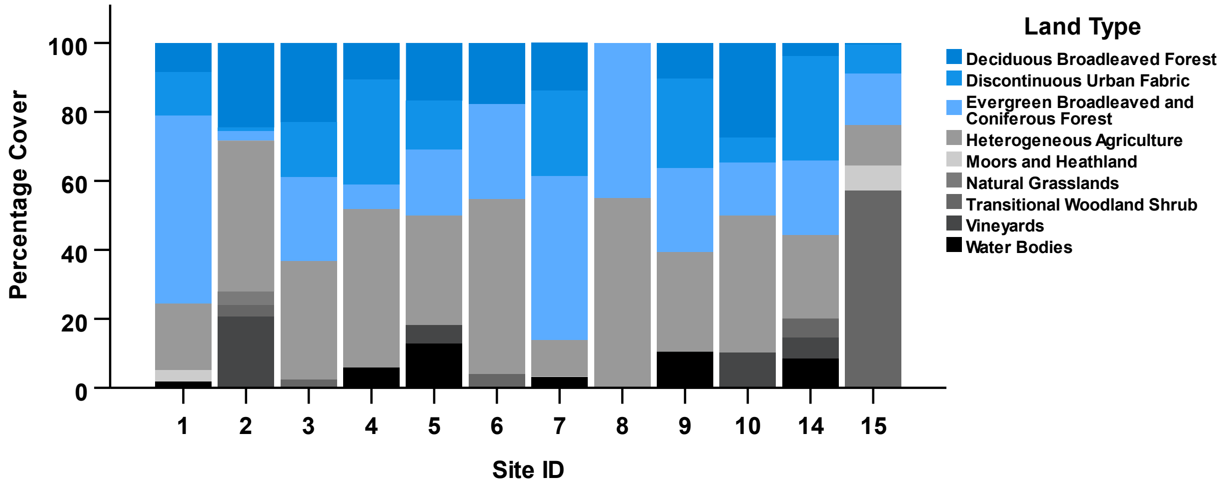

Fig. S2. Land cover percentages at sites (*N*=12). Data was extracted from a 1.5km radius around each site, using the CORINE 2018 land cover dataset in ArcGIS Pro (release v. 3.0). This was then validated against recent satellite imagery to produce modified class definitions that more accurately represented the local vegetation, as detailed in (Table S1).

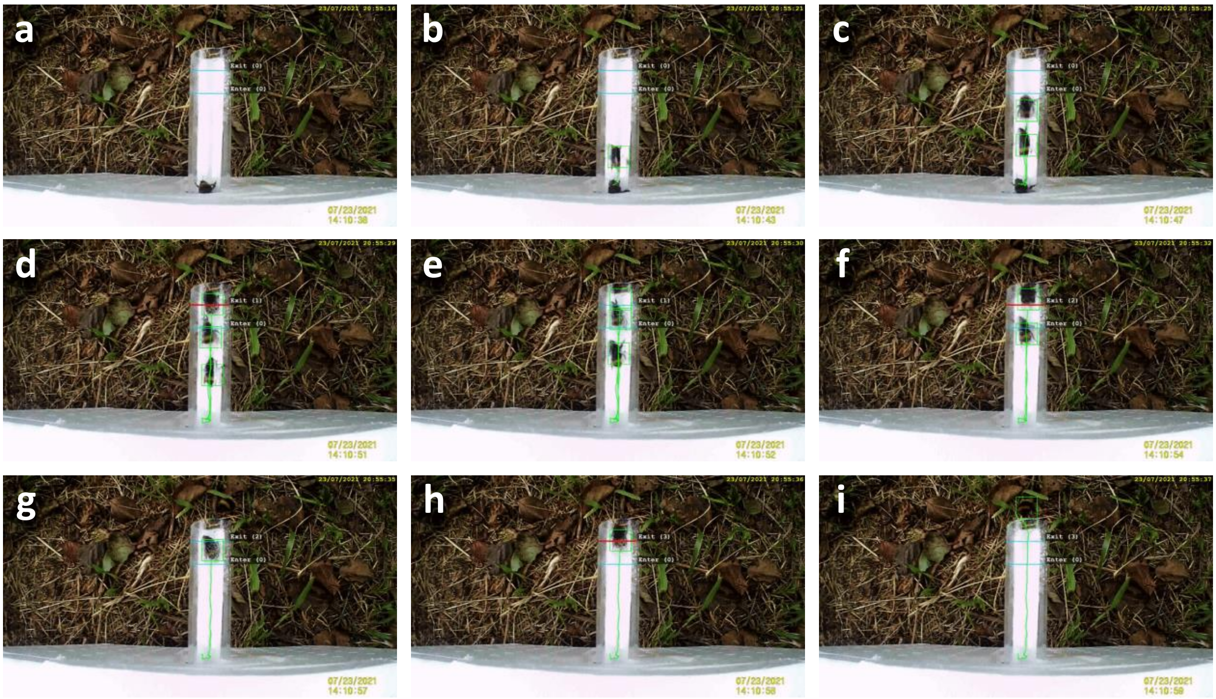

Fig. S3. (a-i) Automated tracking of *B. terrestris* workers exiting a colony. Software was calibrated to an ‘overhead camera’ pre-set, and the detection threshold was set to ‘low’ sensitivity to minimise unwanted noise. The region of interest (ROI) for detection comprised only the entrance tube itself, thus avoiding detections of bees flying nearby which would increase the computational workload. Green boxes indicate individual tracking locks, and green lines denote the resultant tracked trajectories (b-i). Horizontal blue lines designate the digital entrance and exit counters, with each detecting trajectories transecting them in a specific direction (a-i). Resultant detections are logged in the brackets following each label, and indicated by a temporary change in colour from blue to red (d, f, and h). All detections are timestamped and assigned an ID, allowing foraging frequencies over time to be quantified. For full video, see (Supplementary Video 1).

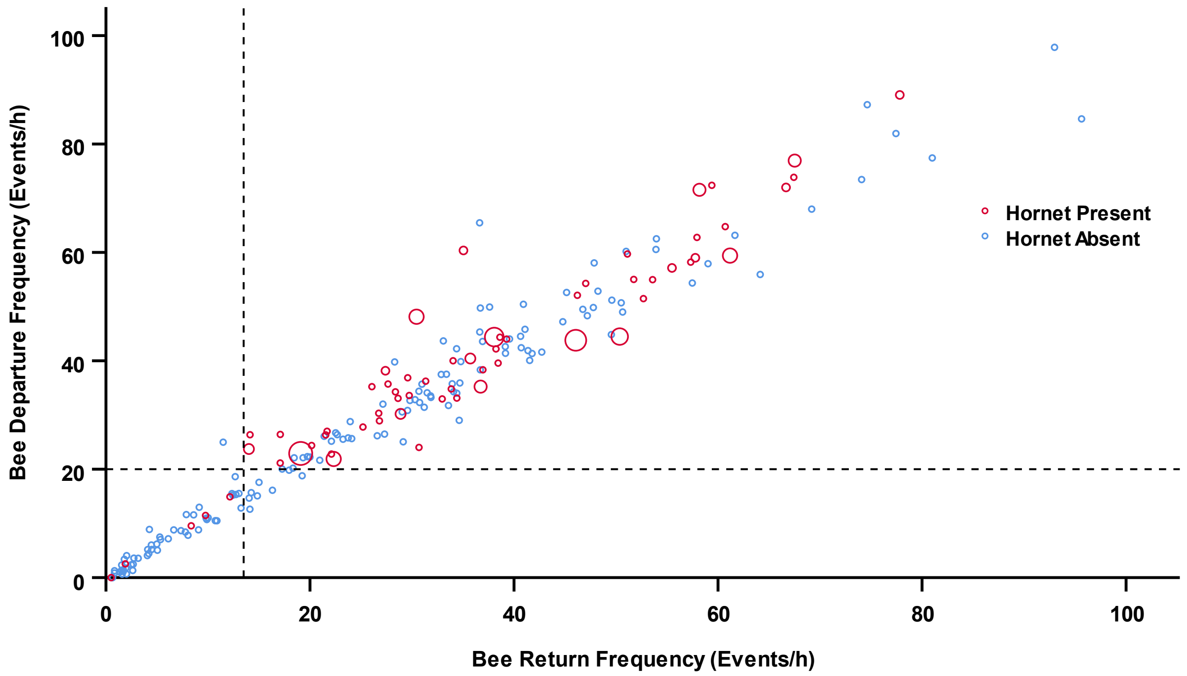

Fig. S4. Frequency of bee departures and returns per hour in relation to hornet presence and predation attempts at colonies (N=36). Each point represents a single sample of bee foraging frequency per hour, coloured based on whether hornets were present or absent at the colony during sampling (absent, blue; present, red). In cases where hornets were present, point size indicates the number of predation attempts observed (larger, greater number of attempts). Dashed lines denote the approximate thresholds above which the majority (>90%) of hornet presence events occurred.

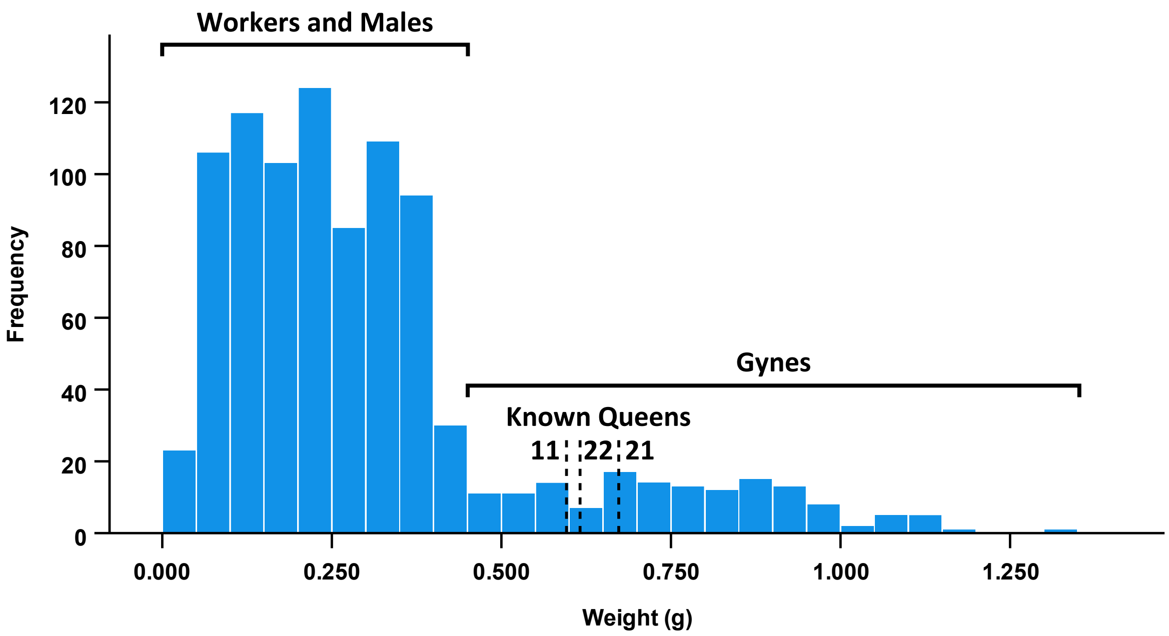

Fig. S5. Adult weight frequency distribution across all colonies (N=940). Brackets indicate bifurcation into two clusters, that of primarily workers and males, and that of gynes. Dashed vertical lines indicate the weight of known queens, and numbers indicate the colony ID to which each belonged. These queens were used to confirm the identity of the heavier low frequency cluster as that of queens and emerging gynes, and the lighter high frequency cluster as that of workers and males. Then, to empirically validate the presence or absence of new gynes in colonies without clearly identifiable queen cells, individual weights were mapped against this frequency distribution. Gynes were classified as individuals weighing ≥0.450g (excluding the heaviest individual in the colony, assumed to be the queen) and thus falling into the gyne weight frequency distribution.

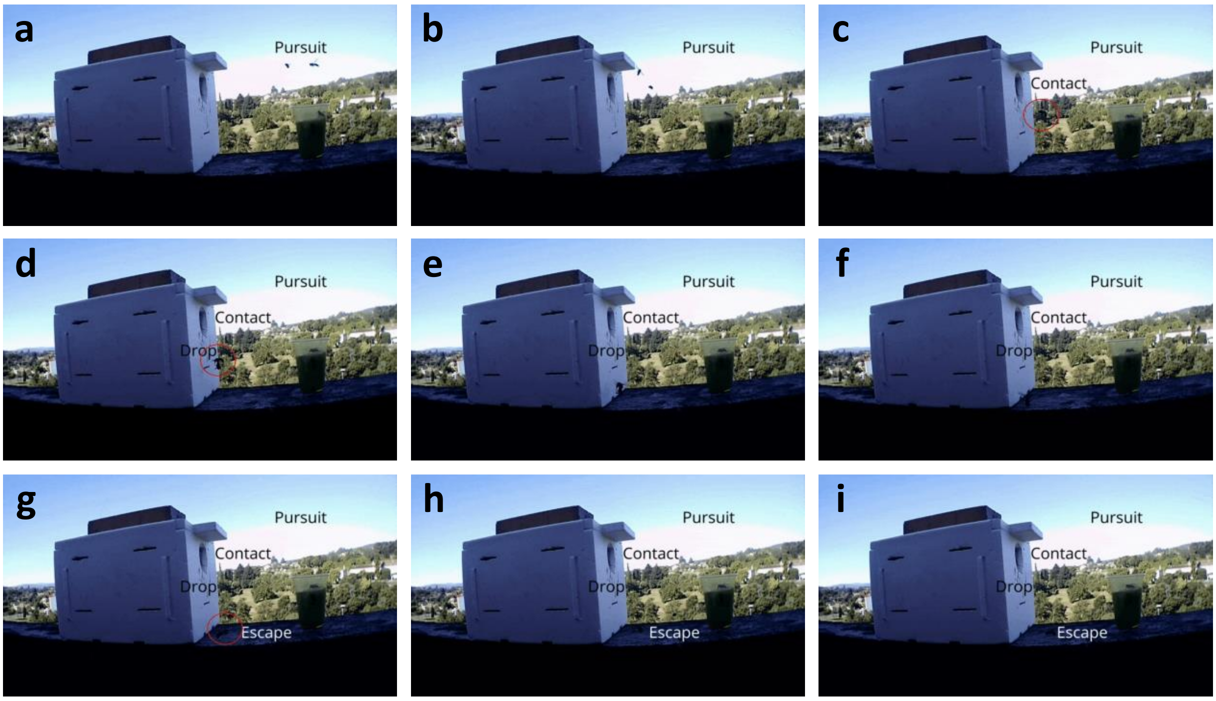

Fig. S6. (a-i) Ethogram of typical predation attempt by *V. velutina* upon *B. terrestris*. This would generally consist of a hornet pursuing a bee (a and b); grabbing the bee during flight (c); dropping to the ground with the bee as it assumed a defensive response (d-f); and upon impact, loosing purchase on the bee, thus allowing it to escape (g-i). Red ovals indicate the timing of key behaviours, and labels denote behaviour type. For full video, see (Supplementary Video 2).

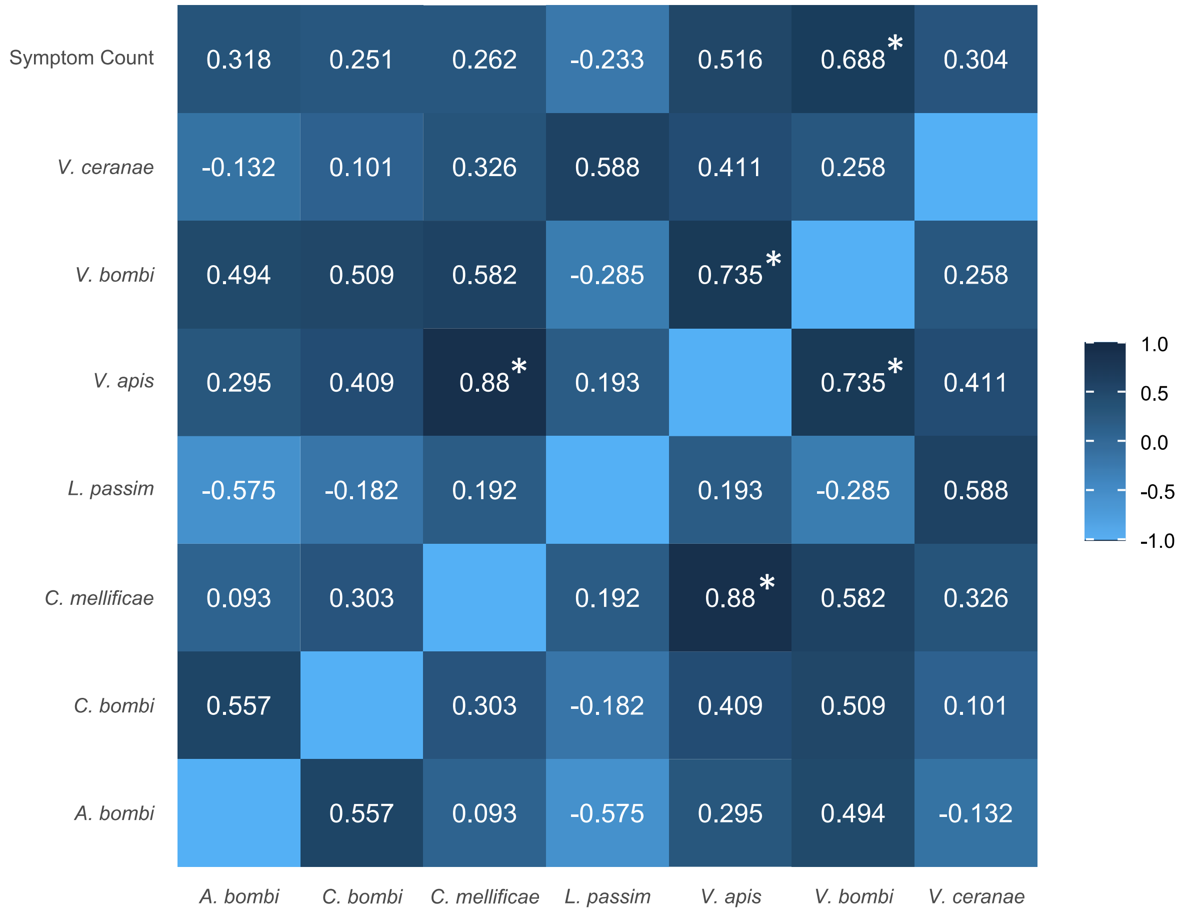

Fig. S7. Heatmap showing Spearman's rank correlations (ρ) between pathogen prevalences and the frequency of observed symptoms for sampled colonies (N=10). Colours indicate the strength of correlations, values denote correlation coefficients, and asterisks designate significance ($P<0.05$).

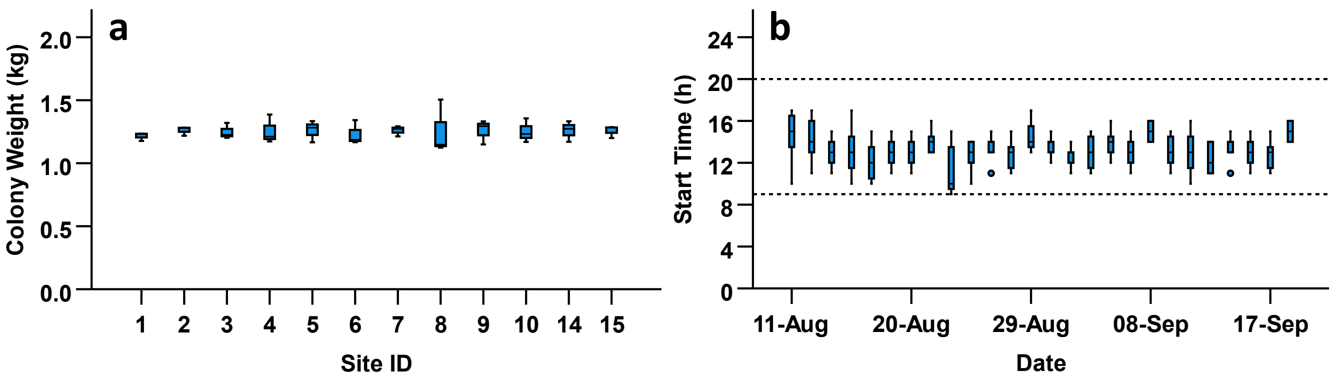

Fig. S8. (a) Starting colony weights grouped by site at the initiation of the experiment (N=36). Weights were normalised to remove the influence of supplementary sucrose solution and waterproof outer coatings. Colony weights did not differ significantly between sites (Kruskal–Wallis Test, χ^2=1.768, N=36, η^2<0.001, P=0.999). (b) Timing of video recordings by day across the course of the study. Dashed lines indicate the window of *B. terrestris* colony foraging activity. Outliers (greater than 1.5 times the interquartile range from the median) are indicated with circles.

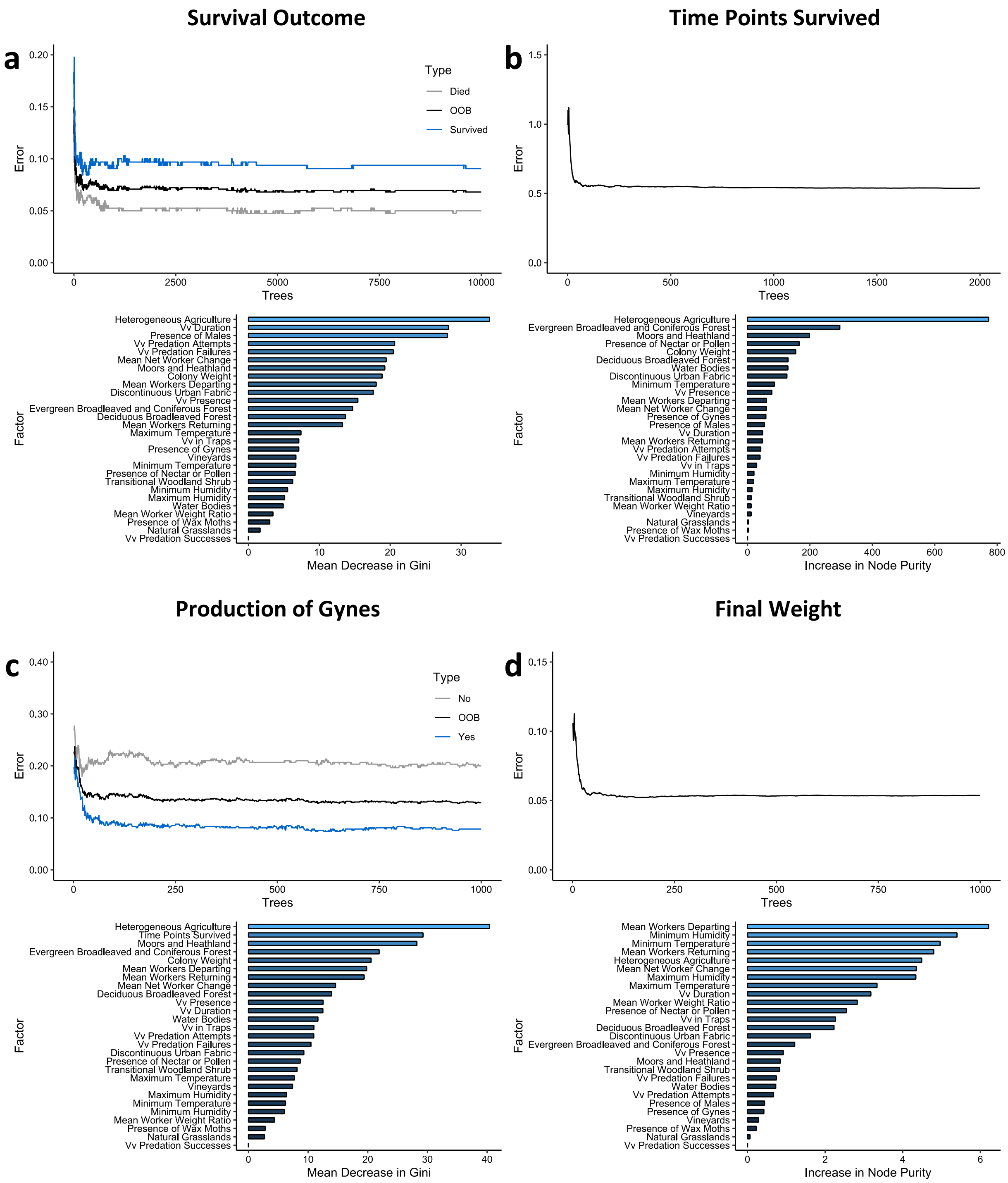
Survival Outcome
a
Type
Died
OOB
Survived
Error
Trees
Factor
Heterogeneous Agriculture
Vv Duration
Presence of Males
Vv Predation Attempts
Vv Predation Failures
Mean Net Worker Change
Moors and Heathland
Colony Weight
Mean Workers Departing
Discontinuous Urban Fabric
Vv Presence
Evergreen Broadleaved and Coniferous Forest
Deciduous Broadleaved Forest
Mean Workers Returning
Maximum Temperature
Vv in Traps
Presence of Gynes
Vineyards
Minimum Temperature
Presence of Nectar or Pollen
Transitional Woodland Shrub
Minimum Humidity
Maximum Humidity
Water Bodies
Mean Worker Weight Ratio
Presence of Wax Moths
Natural Grasslands
Vv Predation Successes
Mean Decrease in Gini
Time Points Survived
b
Error
Trees
Factor
Heterogeneous Agriculture
Evergreen Broadleaved and Coniferous Forest
Moors and Heathland
Presence of Nectar or Pollen
Colony Weight
Deciduous Broadleaved Forest
Water Bodies
Discontinuous Urban Fabric
Minimum Temperature
Vv Presence
Mean Workers Departing
Mean Net Worker Change
Presence of Gynes
Presence of Males
Vv Duration
Mean Workers Returning
Vv Predation Attempts
Vv Predation Failures
Vv in Traps
Minimum Humidity
Maximum Temperature
Maximum Humidity
Transitional Woodland Shrub
Mean Worker Weight Ratio
Vineyards
Natural Grasslands
Presence of Wax Moths
Vv Predation Successes
Increase in Node Purity
Production of Gynes
c
Type
No
OOB
Yes
Error
Trees
Factor
Heterogeneous Agriculture
Time Points Survived
Moors and Heathland
Evergreen Broadleaved and Coniferous Forest
Colony Weight
Mean Workers Departing
Mean Workers Returning
Mean Net Worker Change
Deciduous Broadleaved Forest
Vv Presence
Vv Duration
Water Bodies
Vv in Traps
Vv Predation Attempts
Vv Predation Failures
Discontinuous Urban Fabric
Presence of Nectar or Pollen
Transitional Woodland Shrub
Maximum Temperature
Vineyards
Maximum Humidity
Minimum Temperature
Minimum Humidity
Mean Worker Weight Ratio
Presence of Wax Moths
Natural Grasslands
Vv Predation Successes
Mean Decrease in Gini
Final Weight
d
Error
Trees
Factor
Mean Workers Departing
Minimum Humidity
Minimum Temperature
Mean Workers Returning
Heterogeneous Agriculture
Mean Net Worker Change
Maximum Humidity
Maximum Temperature
Vv Duration
Mean Worker Weight Ratio
Presence of Nectar or Pollen
Vv in Traps
Deciduous Broadleaved Forest
Discontinuous Urban Fabric
Evergreen Broadleaved and Coniferous Forest
Vv Presence
Moors and Heathland
Transitional Woodland Shrub
Vv Predation Failures
Water Bodies
Vv Predation Attempts
Presence of Males
Presence of Gynes
Vineyards
Presence of Wax Moths
Natural Grasslands
Vv Predation Successes
Increase in Node Purity

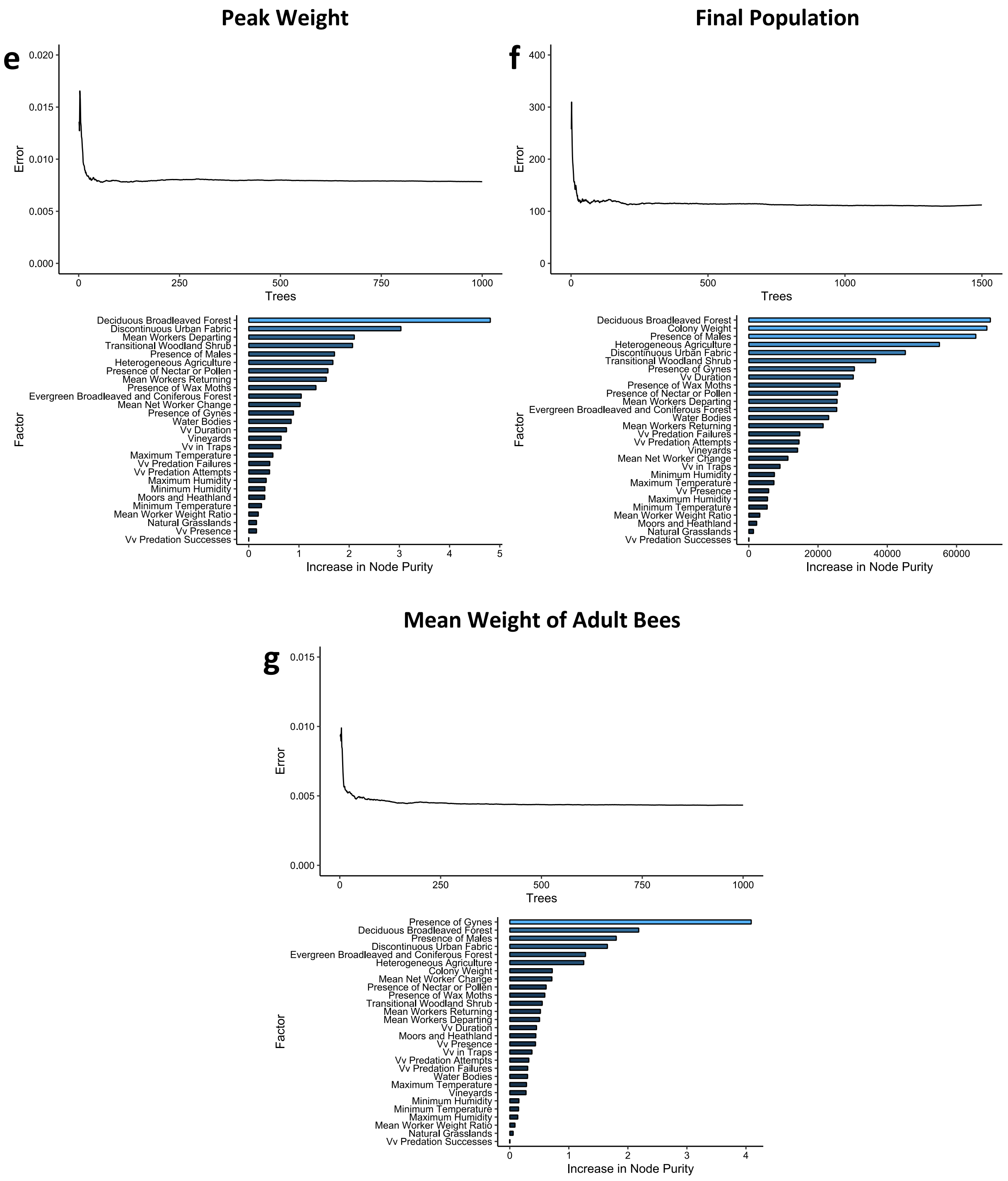

Fig. S9. Error rates and explanatory variable importance rankings for random forest models with response variables of (a) bee colony survival outcome; (b) number of time points for which colonies survived; (c) production of gynes; (d) final colony weight; (e) peak colony weight; (f) final colony population; and (g) mean weight of adult bees. Error rate is displayed by type and total out-of-bag (OOB) error for classification models (a and c), and as a single mean square error (MSE) for regression models (b, d, e, f, and g). Tree numbers for models were optimised via assessment of error rate stabilisation. Importance of explanatory variables is ranked by mean contribution to Gini index in classification models (a and c), and contribution to node purity in regression models (b, d, e, f, and g). With the exception of hypothesis-essential variables, factors following a major drop in Gini index or node purity were excluded from final GLMMs due to a lack of predictive influence.

Table S1. Modified land cover definitions. Classes occupying multiple rows indicate land cover types that have been merged to create a single new modified class. All satellite data were obtained from the ArcGIS Online (release v. 2.25) Esri 2022 satellite imagery WGS84 basemap, at a resolution of ≤1m.

CLC 2018 Class	Modified Class	Description
Discontinuous Urban Fabric	Discontinuous Urban Fabric	Merged to encompass all discontinuous urban areas
Industrial or Commercial Units		
Vineyards	Vineyards	No alteration
Annual Crops Associated with Permanent Crops	Heterogeneous Agriculture	Merged to reflect more general patchwork of agricultural land observed in satellite data
Complex Cultivation Patterns		
Land Principally Occupied by Agriculture with Significant Areas of Natural Vegetation		
Broadleaved Forest	Deciduous Broadleaved Forest	Renamed to specify natural *Quercus* and *Castanea* forests
Coniferous Forest	Evergreen Broadleaved and Coniferous Forest	Merged and renamed as class consisted primarily of *Eucalyptus* and *Pinus*
Mixed Forest		
Natural Grasslands	Natural Grasslands	No alteration
Moors and Heathland	Moors and Heathland	No alteration
Transitional Woodland Shrub	Transitional Woodland Shrub	No alteration
Salt Marshes	Water Bodies	Merged to encompass all water bodies with an area ≥0.1km^2 based on satellite data
Water Courses		
Water Bodies		
Estuaries		

Table S2. List of targets screened for prior to initiation of the study. All viral, bacterial, fungal, acarine, trypanosomal, and neogregarine targets were analysed using standard RT-qPCR protocols developed by the supplier (Biobest Group), and the presence or absence of insect parasites was determined via visual inspection. Conformed absence of all targets was a prerequisite for colonies included in the study.

**Target**	**Class**
ABPV	Viral pathogen
Aethina tumida	Insect parasite
Aphomia sociella	Insect parasite
Apicystis bombi	Neogregarine pathogen
Ascosphaera apis	Fungal pathogen
Aspergillus flavus	Fungal pathogen
Aspergillus fumigatus	Fungal pathogen
Crithidia bombi	Trypanosomal pathogen
CPBV	Viral pathogen
IAPV	Viral pathogen
KBV	Viral pathogen
Kuzinia laevis	Acarine parasite
Locustacarus buchneri	Acarine parasite
Melittobia acasta	Insect parasite
Melittobia chalybii	Insect parasite
Sphaerularia bombi	Nematode parasite
Spiroplasma apis	Bacterial pathogen
Spiroplasma melliferum	Bacterial pathogen
Tropilaelaps clareae	Acarine parasite
Tropilaelaps mercedesae	Acarine parasite
Vairimorpha apis	Fungal pathogen
Vairimorpha bombi	Fungal pathogen
Vairimorpha ceranae	Fungal pathogen
Vitula edmandsii	Insect parasite

Vairimorpha recently reclassified from *Nosema*[1].

Table S3. Summary of pathogen percentage prevalence data for all sampled colonies (*N*=10).

Colony ID	*N*	*V. ceranae*	*V. apis*	*V. bombi*	*C. bombi*	*C. mellificae*	*L. passim*	*A. bombi*	Symptom Count
2	20	0	0	0	15.0	0	5.0	5.0	0
4	20	0	5.0	0	10.0	10.0	5.0	30.0	0
14	14	7.1	28.6	7.1	21.4	14.3	14.3	14.3	2
15	20	5.0	5.0	0	10.0	0	10.0	35.0	1
16	20	0	0	0	5.0	0	0	30.0	0
19	20	0	10.0	15.0	25.0	5.0	0	45.0	5
22	20	5.0	0	5.0	20.0	0	0	35.0	2
25	20	5.0	10.0	10.0	0	10.0	5.0	15.0	2
27	20	5.0	0	0	5.0	0	15.0	0	0
28	20	5.0	10.0	10.0	35.0	10.0	5.0	45.0	0

Vairimorpha recently reclassified from *Nosema*[1].

Table S4. Primer sequence information and annealing temperatures.

Target	Forward sequence	Reverse sequence	Locus	Reference
V. bombi	GGC CCA TGC ATG TTT TTG AAG ATT ATT AT	CTA CAC TTT AAC GTA GTT ATC TGC GG	SSU rRNA	[6]
V. ceranae	CGG CGA CGA TGT GAT ATG AAA ATA TTA A	CCC GGT CAT TCT CAA ACA AAA AAC CG	SSU rRNA	[7]
V. apis	GGG GGC ATG TCT TTG ACG TAC TAT GTA	GGG GGG CGT TTA AAA TGT GAA ACA ACT ATG	SSU rRNA	[7]
C. mellificae	AAT ATT TTA GCA ACT GTG CCT GTA A	ACG ATC ACA AAA ACC ATC AGA GC	cytb	-
L. passim	TTG GCT GAA CRC TTT ATC CC	CAA AAC AAG AGT AAC ACC KCC C	COI	-
C. bombi	TGT ACT TGA GAT TTT ACA GCG TCT	ACA TCA ACT GCT GAA AGC CA	COII	-
A. bombi	GCG CGC TAC ACT GAT ACA C	TTG TCC GTA TTG TTC ACC GGA	SSU rRNA	[8]

Table S5. Thermal protocols.

Target	Taq DNA polymerase activation step	Thermal protocol
V. bombi	10min at 95°C	38 cycles of 30s at 94°C, 30s at 60°C, 10s at 72°C, and 5min at 72°C
V. ceranae	10min at 95°C	38 cycles of 30s at 94°C, 30s at 60°C, 10s at 72°C, and 5min at 72°C
V. apis	10min at 95°C	38 cycles of 30s at 94°C, 30s at 60°C, 10s at 72°C, and 5min at 72°C
C. mellificae	10min at 95°C	35 cycles of 30s at 94°C, 30s at 61°C, 10s at 72°C, and 5min at 72°C
L. passim	10min at 95°C	35 cycles of 30s at 94°C, 30s at 61°C, 10s at 72°C, and 5min at 72°C
C. bombi	10min at 95°C	35 cycles of 30s at 94°C, 30s at 61°C, 10s at 72°C, and 5min at 72°C
A. bombi	10min at 95°C	40 cycles of 30s at 94°C, 30s at 59°C, 10s at 72°C, and 5min at 72°C

Supplementary References

1. Tokarev, Y. S. *et al.* A formal redefinition of the genera Nosema and Vairimorpha (Microsporidia: Nosematidae) and reassignment of species based on molecular phylogenetics. *J Invertebr Pathol* **169**, 107279 (2020).

2. LoCascio, G. M., Aguirre, L., Irwin, R. E. & Adler, L. S. Pollen from multiple sunflower cultivars and species reduces a common bumblebee gut pathogen. *R Soc Open Sci* **6**, (2019).

3. Tritschler, M., Retschnig, G., Yañez, O., Williams, G. R. & Neumann, P. Host sharing by the honey bee parasites Lotmaria passim and Nosema ceranae. *Ecol Evol* **7**, 1850–1857 (2017).

4. Graystock, P., Meeus, I., Smagghe, G., Goulson, D. & Hughes, W. O. H. The effects of single and mixed infections of Apicystis bombi and deformed wing virus in Bombus terrestris. *Parasitology* **143**, 358–365 (2016).

5. Gabín-García, L. B. *et al.* Identification of pathogens in the invasive hornet Vespa velutina and in native Hymenoptera (Apidae, Vespidae) from SW-Europe. *Scientific Reports 2021 11:1* **11**, 1–12 (2021).

6. Plischuk, S. *et al.* South American native bumblebees (Hymenoptera: Apidae) infected by Nosema ceranae (Microsporidia), an emerging pathogen of honeybees (Apis mellifera). *Environ Microbiol Rep* **1**, 131–135 (2009).

7. Martín-Hernández, R. *et al.* Outcome of colonization of Apis mellifera by Nosema ceranae. *Appl Environ Microbiol* **73**, 6331–6338 (2007).

8. Bartolomé, C. *et al.* Longitudinal analysis on parasite diversity in honeybee colonies: new taxa, high frequency of mixed infections and seasonal patterns of variation. *Scientific Reports 2020 10:1* **10**, 1–9 (2020).

Supplementary Methods

Seven specific targets were chosen, consisting of *Vairimorpha ceranae*, *Vairimorpha apis*, *Vairimorpha bombi*, *Crithidia bombi*, *Crithidia mellificae*, *Lotmaria passim*, and *Apicystis bombi* (Table S3). Pathogens were selected based on their high prevalence in nature[1–4], thus best encompassing likely environmental pathogen exposure during the study. Samples were collected from a subset of 10 colonies, consisting of five colonies showing visible symptoms of disease—specifically dysentery—and five showing no visible symptoms. Analyses were conducted using a combination of multiplex and monoplex PCR, and DNA extraction protocols were based on previously established methods[5].

i) DNA Extraction

Samples of 14-20 adult bees were collected from each colony and stored individually in 80% ethanol at 4ºC. Prior to dissection, specimens were washed with 80% ethanol and rinsed three times in sterile distilled H_2O. Guts, Malpighian tubules, and fat bodies were removed and placed into 1.5ml Axygen tubes (Thermo Fisher Scientific) containing 200ml of distilled H_2O, and homogenised utilising disposable plastic pestles (VWR). Per sample, 100µl of the resultant homogenate was added to 300µl of lysis buffer, consisting of 50mM Tris-HCl at pH8, 100mM EDTA, 100mM NaCl, and 1% SDS solution with 5µl of 20mg/ml proteinase K (Thermo Fisher Scientific). This mixture was then left to digest overnight at 56ºC. Genomic DNA was extracted using the phenol-chloroform method, employing a mixture of phenol:chloroform:isoamyl alcohol (Sigma-Aldrich), precipitated with isopropanol, washed in 80% ethanol, and resuspended in 100µl of nuclease-free H_2O (Thermo Fisher Scientific). Following extraction, DNA was standardised to a concentration of ∼70ng/µl, and stored at -20ºC.

ii) Multiplex and Monoplex PCR

PCR amplifications were performed on 2µl aliquots of each sample in a total reaction volume of 15µl, comprising 4.6µl of H_2O, 7.5µl of Multiplex PCR NZYTaq 2x Green Master Mix (NZYTech), and 0.9µl of 5µM primer mix. Two novel triplex reactions were used for *Vairimorpha*, *Crithidia*, and *Lotmaria* primers, while *Apicystis* primers utilised a monoplex reaction. For full primer sequence details, specific marker loci, and thermal protocols, see (Tables S4 and S5). Negative controls were included for each target, consisting of nuclease-free H_2O (Thermo Fisher Scientific). PCR products were separated via 2.5% agarose gel electrophoresis, stained with ethidium bromide, and visualized using a Gel Doc XR UV transilluminator (Bio-Rad).
