# Supplementary material for: Quantifying the impact of an invasive hornet on Bombus terrestris colonies
Source: Commun Biol. 2023 Oct 5;6:990. doi: 10.1038/s42003-023-05329-5 (PMC10556089; doi:10.1038/s42003-023-05329-5)
Supplement: Supplementary file 3 — Description of Additional Supplementary Files [file 42003_2023_5329_MOESM3_ESM.pdf]

Description of Additional Supplementary Files

File name: Supplementary Video 1
Description: Automated tracking of *B. terrestris* workers exiting a colony.

File name: Supplementary Video 2
Description: Ethogram of typical predation attempt by *V. velutina* upon *B. terrestris*.

File name: Supplementary Data
Description: Source data underlying the figures, analyses, and supplementary information.
