# Supplementary figures and images for: Quantifying the impact of an invasive hornet on Bombus terrestris colonies
Source: Commun Biol. 2023 Oct 5;6:990. doi: 10.1038/s42003-023-05329-5 (PMC10556089; doi:10.1038/s42003-023-05329-5)

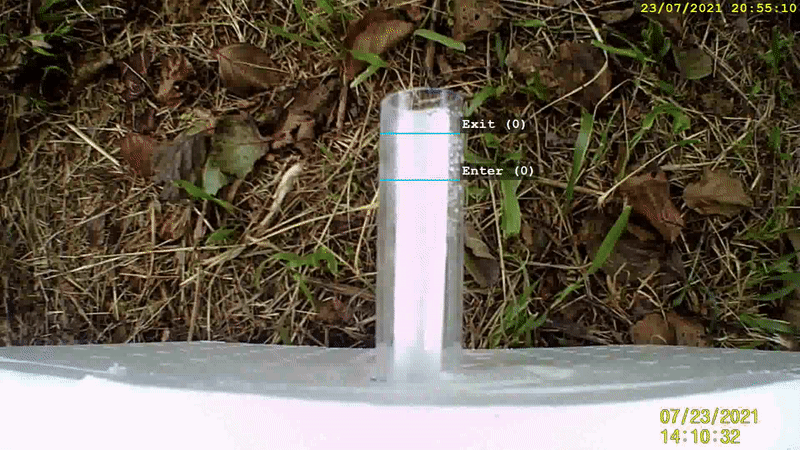

Supplement: Supplementary file 5 — Supplementary Video 1 [file 42003_2023_5329_MOESM5_ESM.gif]

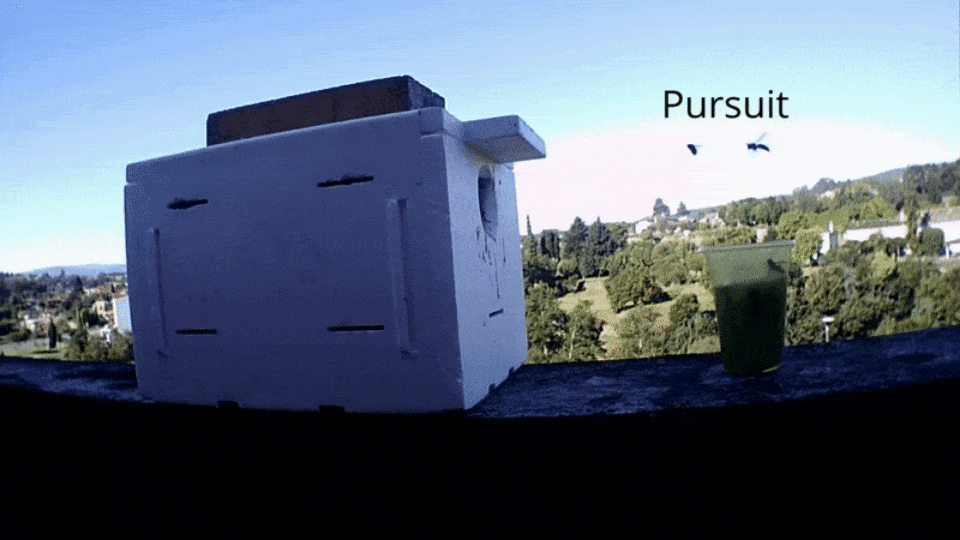

Supplement: Supplementary file 6 — Supplementary Video 2 [file 42003_2023_5329_MOESM6_ESM.gif]
