# Supplementary material for: Quantifying the impact of an invasive hornet on Bombus terrestris colonies
Source: Commun Biol. 2023 Oct 5;6:990. doi: 10.1038/s42003-023-05329-5 (PMC10556089; doi:10.1038/s42003-023-05329-5)
Supplement: Supplementary file 7 — Reporting Summary [file 42003_2023_5329_MOESM7_ESM.pdf]

nature portfolio

Corresponding author(s): Thomas O'Shea-Wheller

Last updated by author(s): 2023/05/31

Reporting Summary

Nature Portfolio wishes to improve the reproducibility of the work that we publish. This form provides structure for consistency and transparency in reporting. For further information on Nature Portfolio policies, see our Editorial Policies and the Editorial Policy Checklist.

Statistics

For all statistical analyses, confirm that the following items are present in the figure legend, table legend, main text, or Methods section.

n/a	Confirmed	
☐	☒	The exact sample size (*n*) for each experimental group/condition, given as a discrete number and unit of measurement
☐	☒	A statement on whether measurements were taken from distinct samples or whether the same sample was measured repeatedly
☐	☒	The statistical test(s) used AND whether they are one- or two-sided<br>*Only common tests should be described solely by name; describe more complex techniques in the Methods section.*
☐	☒	A description of all covariates tested
☐	☒	A description of any assumptions or corrections, such as tests of normality and adjustment for multiple comparisons
☐	☒	A full description of the statistical parameters including central tendency (e.g. means) or other basic estimates (e.g. regression coefficient) AND variation (e.g. standard deviation) or associated estimates of uncertainty (e.g. confidence intervals)
☐	☒	For null hypothesis testing, the test statistic (e.g. *F*, *t*, *r*) with confidence intervals, effect sizes, degrees of freedom and *P* value noted<br>*Give P values as exact values whenever suitable.*
☒	☐	For Bayesian analysis, information on the choice of priors and Markov chain Monte Carlo settings
☐	☒	For hierarchical and complex designs, identification of the appropriate level for tests and full reporting of outcomes
☐	☒	Estimates of effect sizes (e.g. Cohen's *d*, Pearson's *r*), indicating how they were calculated

Our web collection on statistics for biologists contains articles on many of the points above.

Software and code

Policy information about availability of computer code

Data collection	Camlytics (release v .2.2.5), BORIS (release v. 8.5), Tinytag Explorer (release v. 6.0), and ArcGIS Pro (release v. 3.0).
Data analysis	SPSS (release v. 28.0.1.1), and R (release v. 4.2.1).

For manuscripts utilizing custom algorithms or software that are central to the research but not yet described in published literature, software must be made available to editors and reviewers. We strongly encourage code deposition in a community repository (e.g. GitHub). See the Nature Portfolio guidelines for submitting code & software for further information.

Data

Policy information about availability of data

All manuscripts must include a data availability statement. This statement should provide the following information, where applicable:

- Accession codes, unique identifiers, or web links for publicly available datasets
- A description of any restrictions on data availability
- For clinical datasets or third party data, please ensure that the statement adheres to our policy

The authors declare that all supporting data is available within the supplementary information.

Human research participants

Policy information about studies involving human research participants and Sex and Gender in Research.

Reporting on sex and gender	*Use the terms sex (biological attribute) and gender (shaped by social and cultural circumstances) carefully in order to avoid confusing both terms. Indicate if findings apply to only one sex or gender; describe whether sex and gender were considered in study design whether sex and/or gender was determined based on self-reporting or assigned and methods used. Provide in the source data disaggregated sex and gender data where this information has been collected, and consent has been obtained for sharing of individual-level data; provide overall numbers in this Reporting Summary. Please state if this information has not been collected. Report sex- and gender-based analyses where performed, justify reasons for lack of sex- and gender-based analysis.*
Population characteristics	*Describe the covariate-relevant population characteristics of the human research participants (e.g. age, genotypic information, past and current diagnosis and treatment categories). If you filled out the behavioural & social sciences study design questions and have nothing to add here, write "See above."*
Recruitment	*Describe how participants were recruited. Outline any potential self-selection bias or other biases that may be present and how these are likely to impact results.*
Ethics oversight	*Identify the organization(s) that approved the study protocol.*

Note that full information on the approval of the study protocol must also be provided in the manuscript.

Field-specific reporting

Please select the one below that is the best fit for your research. If you are not sure, read the appropriate sections before making your selection.

☐ Life sciences ☐ Behavioural & social sciences ☒ Ecological, evolutionary & environmental sciences

For a reference copy of the document with all sections, see nature.com/documents/nr-reporting-summary-flat.pdf

Ecological, evolutionary & environmental sciences study design

All studies must disclose on these points even when the disclosure is negative.

Study description	The study aimed to assess the effect of differing Vespa velutina densities upon Bombus terrestris colonies in the field. To achieve this, 36 B. terrestris colonies were distributed across 12 sites, with three such colonies per site. Each site varied in ambient V. velutina densities, thus allowing the quantification of effects upon B. terrestris colony health, behaviour, and survival. All colonies were sampled every two days across a 40-day period.
Research sample	36 commercial Bombus terrestris colonies purchased from Biobest group, situated across 12 sites within the province of Pontevedra, Spain.
Sampling strategy	Colony health measures were sampled for all colonies across 20 time points, colony foraging behaviour was sampled across 13 time points, and hornet behaviour was sampled across 6 time points. Requisite sample sizes were determined using standard deviation (σ) and mean difference (δ) values, to provide a minimum power ($1-\beta$) of 0.80, at an alpha (α) of 0.05.
Data collection	T.A.O.-W, R.J.C, E.K.J.G, D.S.R, and P.J.K, collected the data. Colony health measurements and hornet trap counts were recorded manually, while colony foraging behaviour and hornet behaviour were recorded remotely using video cameras. Additionally, climatic conditions were recorded remotely using data loggers, and land cover data was obtained from satellite imagery.
Timing and spatial scale	Experimental sampling was initiated on the 9th of August 2021, and concluded on the 18th of September 2021. Colony health measures were sampled across 20 evenly dispersed intervals, while foraging behaviour and hornet behaviour were sampled across 13 and 6 evenly dispersed intervals respectively. These latter two measurements were constrained by the need to deploy, retrieve and recharge cameras, hence their larger sampling intervals. All sampling occurred at sites distributed across the province of Pontevedra, Spain, encompassing an area of ~20km2.
Data exclusions	Data from colonies that were no longer alive was excluded from analyses. This was collected for descriptive purposes, however the decision to exclude such data was made a priori.
Reproducibility	Three additional Bombus terrestris colonies were used to test experimental and methodological reproducibility prior to and during the study.
Randomization	All Bombus terrestris colonies were weighed and assigned a random ID number using the Mersenne Twister algorithm. These were then randomly allocated site ID numbers in groups of three, before making the minimum number of reassignments in order to equalise colony weights across sites. This process was used to ensure an even starting weight distribution across sites, while enabling randomisation within these limits.

Blinding	Blinding in data collection at field sites was achieved by sampling allocation ensuring that no one recorder was aware of both the hornet density and colony health metrics simultaneously. Blinding during video data collection and analyses was ensured via usage of the randomised colony and site ID numbers.

Did the study involve field work? ☒ Yes ☐ No

Field work, collection and transport

Field conditions	Fieldwork occurred in Pontevedra, Spain, from the 9th of August 2021, to the 18th of September 2021. Field sites were located across a patchwork of natural and agricultural land cover, in order to encompass habitat variation at the landscape-scale. Average daily temperatures were ~25°C, and average monthly rainfall was ~59mm.
Location	Pontevedra province, Spain, 42.134000, -8.677000 (DD).
Access & import/export	Importation of relevant equipment was in compliance with EU customs regulation, and all biological samples remained within Spain. Permission to conduct the work was obtained from all private and commercial land owners on whose property field sites resided, prior to study initiation.
Disturbance	The establishment of Bombus terrestris colonies at sites posed a potential disturbance hazard to humans. To mitigate this, we informed land owners of the appropriate behaviour around colonies, and ensured that all colonies were placed in secluded locations at a safe distance from human activity.

Reporting for specific materials, systems and methods

We require information from authors about some types of materials, experimental systems and methods used in many studies. Here, indicate whether each material, system or method listed is relevant to your study. If you are not sure if a list item applies to your research, read the appropriate section before selecting a response.

Materials & experimental systems

n/a	Involved in the study	
☒	☐	Antibodies
☒	☐	Eukaryotic cell lines
☒	☐	Palaeontology and archaeology
☐	☒	Animals and other organisms
☒	☐	Clinical data
☒	☐	Dual use research of concern

Methods

n/a	Involved in the study	
☒	☐	ChIP-seq
☒	☐	Flow cytometry
☒	☐	MRI-based neuroimaging

Animals and other research organisms

Policy information about studies involving animals; ARRIVE guidelines recommended for reporting animal research, and Sex and Gender in Research

Laboratory animals	Bombus terrestris terrestris colonies, containing a queen and ~80 workers, purchased from Biobest group based in in Almeria, Spain. Colonies were screened by the supplier for a panel of common pathogens and parasites using RT-qPCR, to confirm the absence of disease prior to establishment in the field.
Wild animals	Vespa velutina were observed and trapped in the field, traps contained foam inserts to limit fatality, and all V. velutina and other insects trapped were released upon assessment. Additional, V. velutina workers were captured in sample tubes for weighing, and then released following weight data collection.
Reporting on sex	For Bombus terrestris, sex was considered only in the context of colony reproductive output, as queens and males constitute a minority of individuals in colonies. Males were identified via extrusion of the endophallus, while queens were differentiated by mapping their position relative to known queens in the overall weight (g) frequency distribution of colonies. For Vespa velutina, sex was considered in the context of worker weights, to ensure that males were not sampled. This was achieved visually via assessment of the antennae.
Field-collected samples	Bombus terrestris samples used in pathogen analyses were collected from each colony and stored individually in 80% ethanol at 4ºC. Prior to dissection, specimens were washed with 80% ethanol and rinsed three times in sterile distilled H2O. All colonies were removed from field sites at the conclusion of experiments, and disposed of at the University of Vigo.
Ethics oversight	Guidance was provided by the University of Exeter Research Ethics and Governance team, and the Ethical Advisory Board at Universidade de Vigo. Specifically, the study received ethics approval from the University of Exeter, and was assigned the application

reference: eCORN000212 v2.0.

Note that full information on the approval of the study protocol must also be provided in the manuscript.
